# Texture analysis of [^18^F]FDG PET/CT may stratify risk in stage II colorectal cancer – discovery findings

**DOI:** 10.1007/s00259-025-07568-6

**Published:** 2025-10-06

**Authors:** Louise Rice, Balaji Ganeshan, Simon Wan, Shih-hsin Chen, David M.L. Lilburn, Manuel Rodriguez-Justo, Stuart Taylor, Robert I. Shortman, Raymond Endozo, Saif Khan, Luke R. Hoy, Daren Francis, Tan Arulampalam, Nicholas Reay-Jones, Kenneth A. Miles, Ashley M. Groves

**Affiliations:** 1 Institute of Nuclear Medicine, Division of Medicine, University College London (UCL), London, UK; 2 Research Department of Targeted Intervention, Division of Surgery and Interventional Science, University College London (UCL), London, UK; 3 Department of Nuclear Medicine, Keelung Chang Gung Memorial Hospital, Keelung, Taiwan; 4 PET Centre, School of Biomedical Engineering & Imaging Sciences, St Thomas’ Hospital, King’s College London (KCL), London, UK; 5 Surgery and Cancer Board, Imaging Division, University College Hospital (UCH), University College London Hospitals (UCLH) NHS Foundation Trust, London, UK; 6 Centre of Medical Imaging, Division of Medicine, University College London (UCL), London, UK; 7 Department of Colorectal Surgery, Barnet and Chase Farm Hospitals, Royal Free London NHS Foundation Trust, London, UK; 8 Department of Surgery, Department of Clinical Oncology, Colchester General Hospital, East Suffolk and North Essex NHS Foundation Trust, Essex, UK; 9 Colorectal Surgery, Queen Elizabeth II Hospital, East and North Hertfordshire NHS Trust, Hertfordshire, UK; 10 Institute of Nuclear Medicine, University College London Hospital (UCLH), London, UK

**Keywords:** [^18^F]FDG PET/CT, Colorectal cancer, CT texture, Survival

## Abstract

**Rationale:**

It is challenging to identify which patients with Stage II colorectal cancer (T3N0M0 and T4N0M0) are at high risk of recurrence and might benefit from additional therapies. This preliminary study examines whether multiparametric [^18^F]FDG PET/CT is superior to T-stage in predicting overall survival in this patient group.

**Materials and methods:**

This multicentre, prospective observational study included 66 patients (41 male, 25 female, mean age 69.1 ± 10.3yrs) with biopsy-proven Stage II colorectal cancer who underwent [^18^F]FDG PET/CT prior to resection. Kaplan-Meier analysis was performed on PET and image texture parameters and clinico-histopathological markers to identify associations with survival. P-values were adjusted using the Benjamini-Hochberg procedure, and the most statistically significant radiomic parameters underwent 3-fold cross-validation. Multivariate Cox regression analysis was used to determine independence of prognostic markers.

**Results:**

49 patients survived the follow up period, with a mean overall survival of 88.4 (± 42.3 months). Univariate analysis showed no significant survival association with clinical variables such as patient sex (*p* = 0.295), age (*p* = 0.085), tumour side (*p* = 0.662), location (*p* = 0.848) or volume (*p* = 0.782), or high-risk histopathological metrics including low number lymph node sampling (*p* = 0.363), perineural, lymphovascular or extramural tumour invasion (*p* = 0.196), poor tumour differentiation (*p* = 0.372), and tumour perforation (*p* = 0.475). No significant survival difference was observed between T3 and T4-stages (*p* = 0.748). An increased mortality risk was observed for patients with a pixel mean intensity < 20.390 on CT texture (coarse texture scale), HR: 3.40 (1.36–8.49); *p* = 0.005, and where SUVmax ≥ 25.560 g/mL on [^18^F]FDG PET/CT, HR: 3.45 (1.31–9.12); *p* = 0.008. These parameters remained significant after 3-fold cross validation and were independent predictors of survival after Multivariate Cox regression analysis. A higher mortality risk was indicated when the parameters were combined (5 of 66 patients), hazard ratio: 5.44 (1.75–16.91); *p* = 0.001.

**Conclusion:**

Multiparametric [^18^F]FDG PET/CT can potentially provide prognostic markers for patients with stage II colorectal cancer with superior risk stratification compared to T-stage.

## Introduction

Colorectal cancer is the third most common cancer diagnosed globally, and the second most common cause of cancer-related death [1, 2]. Approximately 40% of patients present with stage II disease for which surgical resection is the cornerstone of patient management. Five-year survival in those with localised disease currently ranges from 65 to 85%, indicative of a highly heterogenous disease [3]. Whilst the survival benefits of adjuvant therapy in stage III - or greater - colon cancer (or neoadjuvant therapy in the case of rectal cancer) have been convincingly demonstrated, those presenting with node-negative disease represent a clinical dilemma in terms of optimum patient management [4–6].

To date, trials of adjuvant therapy in stage II colon cancer have not demonstrated a clear overall survival or disease-free survival benefit [7, 8]. Preoperative radiotherapy or chemoradiotherapy has been shown to decrease local recurrence rates in stage II/III rectal cancers, however it is associated with significant rates of intestinal and sexual morbidity postoperatively [6]. Decisions regarding neoadjuvant and adjuvant treatment tend to be made on an individual basis, with reference to high-risk clinicopathological and molecular features such as higher T-stage, bowel obstruction at presentation, microsatellite instability/mismatch repair deficiency, and fewer than 12 surgically sampled lymph nodes for histological examination [4]. Nonetheless, even within this higher risk subgroup, the overall survival benefit in colon cancer from adjuvant therapy is modest at best. A significant proportion of stage II ‘higher risk’ patients who have their tumours resected without adjuvant or neoadjuvant treatment do not have disease recurrence, indicating that some patients within this stratum are being overtreated. Similarly, a considerable proportion of patients with lower risk clinicopathological features have cancer recurrence and lower overall survival, indicating that they might benefit from more aggressive treatment [3, 7, 9]. Improved strategies are required to better select those patients who are at high risk of recurrence and who are likely to derive the most benefit from neoadjuvant/adjuvant treatment, and those patients at lower risk who may best be managed with surgery and close follow up alone. Risk stratification is critical to achieve the optimal balance in improving patient survival while avoiding chemotherapy and radiotherapy side-effects where such treatment confers no benefit [9].

Identification of an imaging signature for high-risk stage II colorectal cancers has the potential to complement histology findings and better guide adjuvant management of patients. Imaging can provide prognostic information at an earlier stage on the clinical pathway compared to histological markers [10]. The ability to assess the tumour in its entirety could circumvent the limitation of biopsy under sampling, potentially allowing for more comprehensive characterisation of tumour phenotype and prediction of tumour behaviour. This ability to risk stratify can further guide multi-disciplinary decisions regarding patient management.

Fluorine-18 Fluorodeoxyglucose ([^18^F]FDG) Positron Emission Tomography/Computed Tomography (PET/CT) is an imaging technique which is currently used in the pre-operative staging of selected patients with colorectal cancer [11]. It has been demonstrated that increased tumour uptake of [^18^F]FDG, quantified as the maximum Standardized Uptake Value (SUVmax), negatively correlates with overall survival with lower radiotracer uptake being associated with increased patient survival [12]. Furthermore, tumours which demonstrate a lower Metabolic Tumour Volume (MTV) on [^18^F]FDG PET/CT have been shown to be associated with increased patient survival in a study involving 231 participants [13].

Additional prognostic information can be gleaned from the CT component of [^18^F]FDG PET/CT through texture analysis, a radiomic process which attempts to quantify heterogeneity within digital images via measurement of pixel values within a given region of interest [14]. Prior studies have shown a correlation between CT texture analysis (CTTA) and tumour characteristics such as hypoxia and angiogenesis, identifying lesions which are more biologically aggressive [15]. CTTA findings have previously been shown to correlate with survival and/or treatment response in colorectal cancer [16, 17], small cell lung cancer [18], hepatocellular carcinoma [19], renal cell carcinoma [20], neuroendocrine tumours [21], and inflammatory breast cancer [22]. It is therefore envisaged that imaging techniques which combine [^18^F]FDG PET/CT and CTTA imaging biomarkers could be used to improve prognostic stratification and treatment decisions for patients.

This preliminary study aims to determine whether multiparametric [^18^F]FDG PET/CT incorporating CTTA is superior to T-stage in the prediction of overall survival for patients with stage II colorectal cancer.

## Materials and methods

### Study design and patient cohort

This prospective, observational study forms part of a larger cohort of 421 patients with colorectal cancer. This study was approved by the London-Harrow Research Ethics Committee and all participants signed an informed consent form. Patients with biopsy-proven colorectal cancer were recruited from 9 local hospitals. All the study sub-population (66 participants) had an [^18^F]FDG PET/CT scan, before proceeding to primary surgical resection of the tumour. The resected surgical specimen was confirmed at histology to be adenocarcinoma in all cases. All decisions regarding the patient’s clinical management were made by the local multi-disciplinary team (MDT), and patients were followed up at their local hospital.

In total, 66 patients out of the cohort of 421 were found to be at intermediate risk as indicated by TNM Stage II, specifically T3N0M0 and T4N0M0 disease on clinical and pathological stage. The primary end point of the study was overall survival, defined as the time between the date of the [^18^F]FDG PET/CT scan and the date of death as determined from review of clinical records. Patients were censored for all time points beyond the follow-up period of May 2023.

### Image acquisition

All [^18^F]FDG PET/CT scans were performed on a GE Discovery VCT 64 PET/CT scanner (General Electric Healthcare, Amersham, UK) at a single institution. Patients were instructed to fast for at least 4 h prior to an injection of 250 +/- 80 MBq of [^18^F]FDG and were scanned after 66 +/- 7 min of uptake time. A low dose, unenhanced CT scan was acquired from the skull vertex to the upper thighs for attenuation correction and anatomical reference (120 kVp, 60 mAs, 64 × 0.6 mm detectors; standard reconstruction soft tissue kernel and a filtered-back projection algorithm). This was followed by a PET scan acquired in 2D mode at 4 min/bed position, with identical anatomical coverage. PET images were reconstructed using Ordered Subsets Expectation Maximisation (OSEM) with 2 iterations and 28 subsets, 3.27 mm slice thickness. The PET voxel size was 5.41 mm x 5.41 mm x 3.27 mm and the reported axial and radial spatial resolution of the scanner at centre of field of view was 4.7 mm and 4.2 mm, respectively.

### 18 F-FDG PET/CT analysis

The PET emission study and CT scan were viewed independently and co-registered using the PET Volume Computer Assisted Reading program on a GE Advantage Workstation (General Electric Healthcare, Milwaukee, Wisconsin, USA) by a nuclear medicine physician with > 5 years of experience in oncological imaging. A measurement box was manually placed around the tumour and adjusted to avoid adjacent regions of high physiological tracer activity (e.g. bladder). A volume of interest (VOI) was automatically segmented within the box using a 40% threshold relative to the voxel value with most intense tumour uptake (defined as SUVmax measured in g/mL). Total lesion glycolysis (TLG) was defined as the average SUV intensity of all the voxels in the VOI multiplied by the total volume of voxels included in the VOI.

### Texture analysis

Texture analysis was performed using a commercially available research software called TexRAD (Feedback Medical Ltd., London/Cambridge, UK). The software has an embedded DICOM viewer and is installed as a server-web-client solution within the hospital network, communicating via DICOM protocol with the PACS or data-archive (mini-PACS) to receive DICOM images for analysis. The following process was undertaken exclusively within the TexRAD software.

Regions of interest (ROIs) were manually drawn around the tumour at the most metabolically active axial slice on both the [^18^F]FDG PET images and the CT images. The ROIs were drawn by a medical doctor with 3-years’ experience of drawing tumour ROIs on PET and CT scans, under the supervision of an imaging researcher with 7 years’ experience in CTTA, and a dual-accredited radiologist/nuclear medicine specialist with 10 years of PET/CT experience. The CTTA technique used is described in detail in a previous study [23]. A threshold based on Hounsfield units was applied to all pixels within the ROI to exclude air (<−50HU) and calcification/bone (> 200HU). This was followed by a filtration-step comprising of a series of bandpass Laplacian of Gaussian filters corresponding to spatial scale filter (SSF) at 0, 2, 3, 4, 5 and 6 mm (radii). This step extracted and enhanced image features of specific sizes and intensities within the ROI, where SSF = 0 corresponded to an unfiltered image, SSF = 2 corresponded to a fine texture map, SSF = 3–5 corresponded to a medium texture map and SSF = 6 corresponded to a coarse texture map. Quantification of texture within the unfiltered image and each texture map comprised the following statistical and histogram-based metrics: mean, standard deviation, entropy (higher values reflecting irregularity), mean of positive pixels, skewness (where a positively skewed histogram distribution reflects a preponderance of bright objects) and kurtosis (where high values reflect high tissue contrast). [^18^F]FDG PET texture analysis (PTA) comprised quantification of the same texture metrics above without filtration owing to the inherent poorer resolution of PET images.

**Fig. 1:**
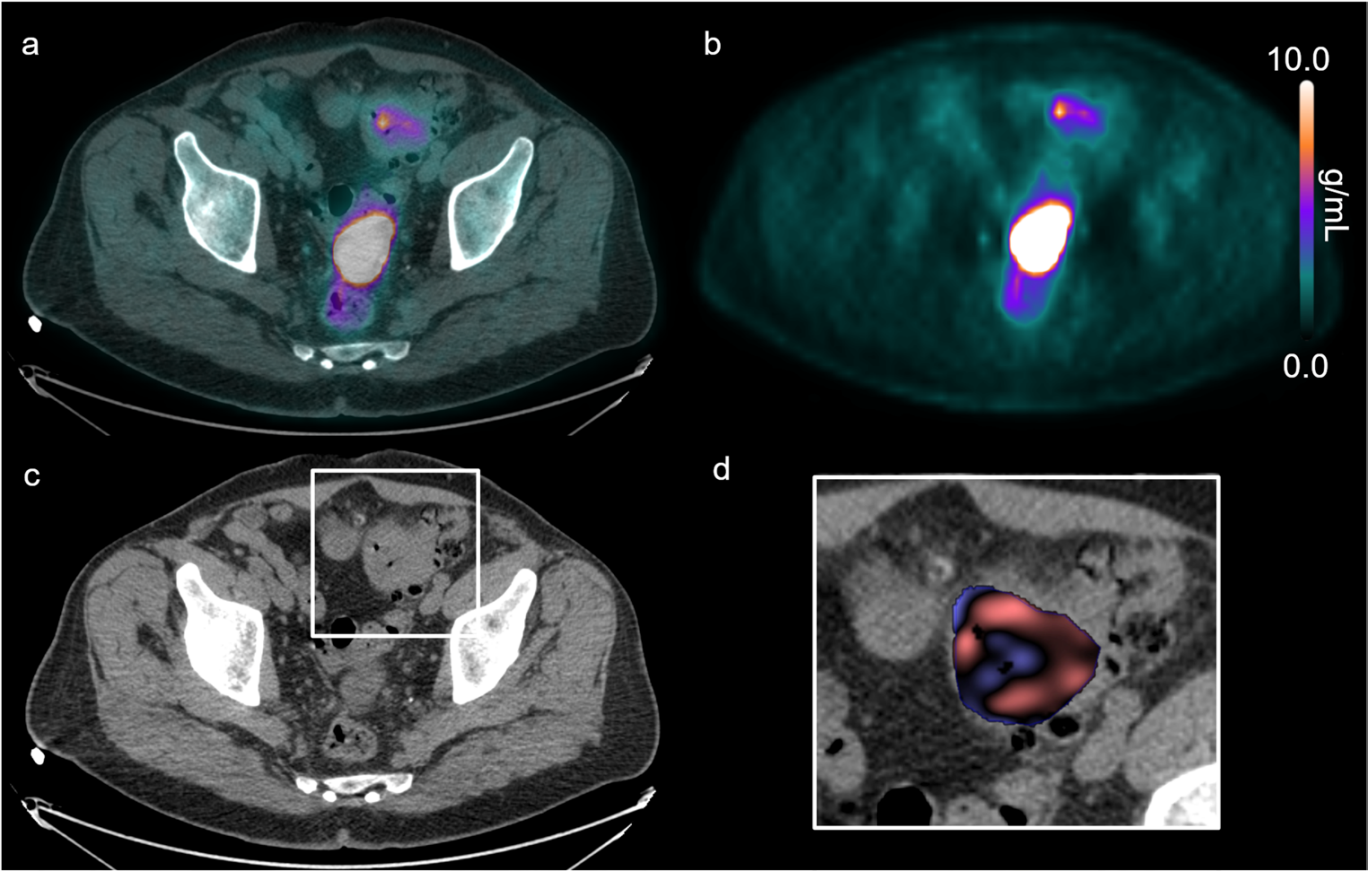
[^18^F]FDG PET/CT imaging and CT texture analysis of a patient in the poor survival group. Co-registered [^18^F]FDG PET/CT (**a**) and PET emission scan (**b**) showing a relatively high tumour SUVmax. Corresponding axial CT slice displaying the tumour (white box) (**c**) and enlarged CT view of the tumour with a superimposed CT texture map showing a low mean intensity at coarse texture-scale (**d**)

**Fig. 2:**
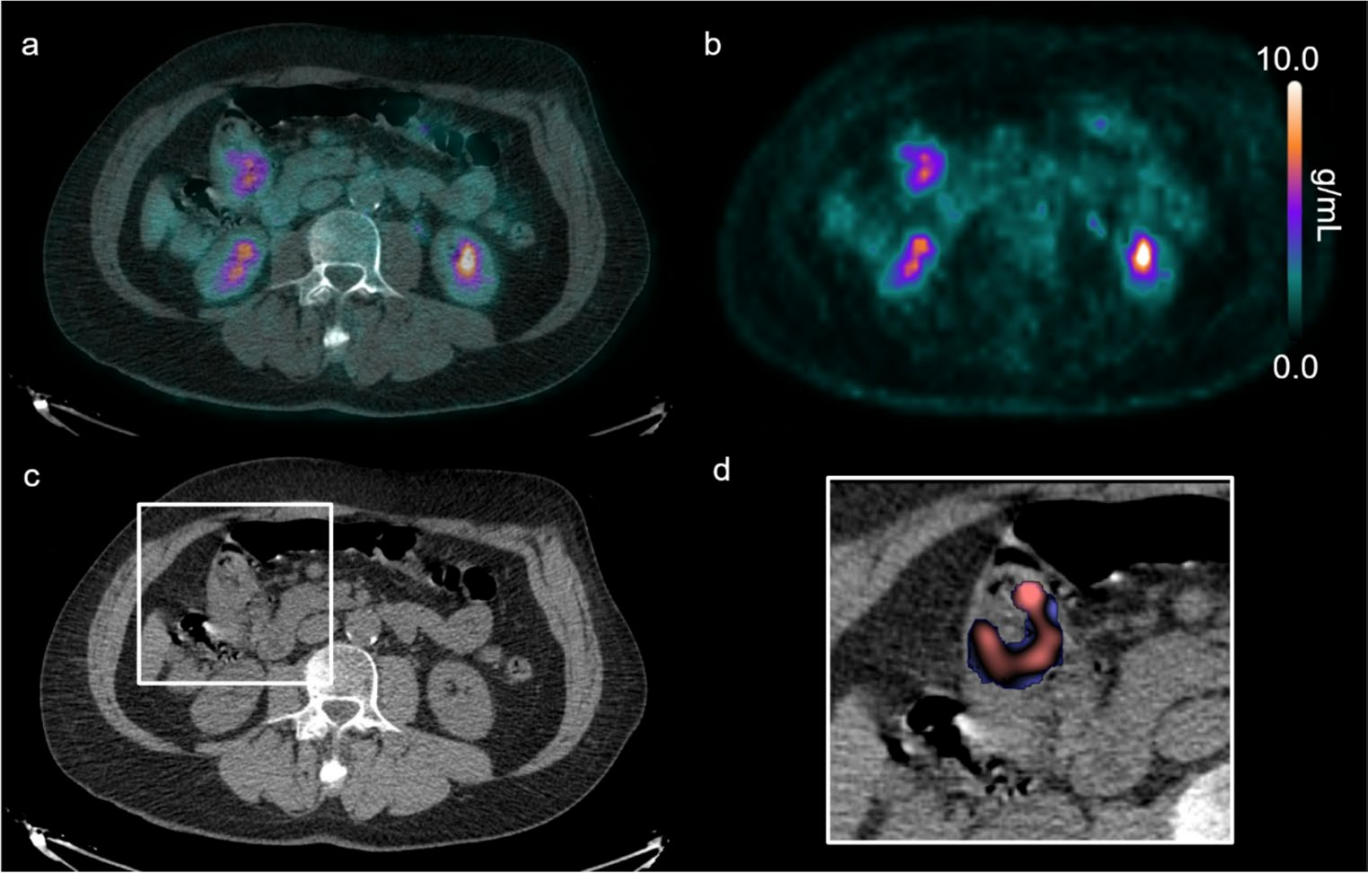
[^18^F]FDG PET/CT imaging and CT texture analysis of a patient in the good survival group. Co-registered [^18^F]FDG PET/CT (**a**) and PET emission scan (**b**) showing a relatively low tumour SUVmax. Corresponding axial CT slice displaying the tumour (white box) (**c**) and enlarged CT view of the tumour with a superimposed CT texture map showing a high mean intensity at coarse texture-scale (**d**)

Figures 1 and 2 show representative [^18^F]FDG PET/CT imaging and CTTA for patients in both poor and good prognostic groups, respectively.

### Histopathological analysis

Pathological risk factors, including poor tumour differentiation, tumour perforation, sampling of fewer than 12 lymph nodes and perineural/lymphovascular/extramural venous invasion (EMVI) were obtained from historic pathological reports, reported and reviewed by expert histopathologists [24]. This information was available for the majority of patients, but there was missing data in some cases (data is available in 57 cases for number of lymph nodes sampled, 60 cases for EMVI, 56 cases for lymphovascular and/or perineural invasion, and 55 cases for tumour perforation or its absence, out of the 66 patients in our cohort).

### Statistical analysis

The ability of [^18^F]FDG PET/CT, CTTA, PTA and T-stage to help predict overall survival was assessed using univariate optimised Kaplan-Meier (KM) survival analysis. KM analysis used an iterative method to identify the optimal threshold for each parameter that best separated patients into good and poor prognostic groups (indicated by the most significant p-value from Log-rank test). To address the multiple survival comparisons arising from the above step, a Benjamini-Hochberg correction was employed to control the false discovery rate at 0.2. Where several parameters were found to be significant with equivalent p-values, the best parameters were those which had a similar proportion of patients in good and poor prognostic groups. Additionally, markers which were also found to be significant at the median cutoff level were chosen as this showed the parameter to be more robust, as it was considered an unbiased estimation of hazard.

3-fold cross-validation was undertaken for the best significant markers after Benjamini-Hochberg correction, with 3 folds having proportional numbers of events and non-events (66 patients with 19 events). Cutoffs were established in 2 out of the 3 folds and tested in the remaining fold. Patients with poor and good prognosis defined for each validation subgroups were re-combined to stratify the complete cohort, which was then assessed using KM survival analysis to determine statistical significance after cross-validation.

Multivariate Cox regression analysis was undertaken by developing a model incorporating the markers which remained significant after 3-fold cross validation to determine which parameters were independent predictors of survival.

**Fig. 3:**
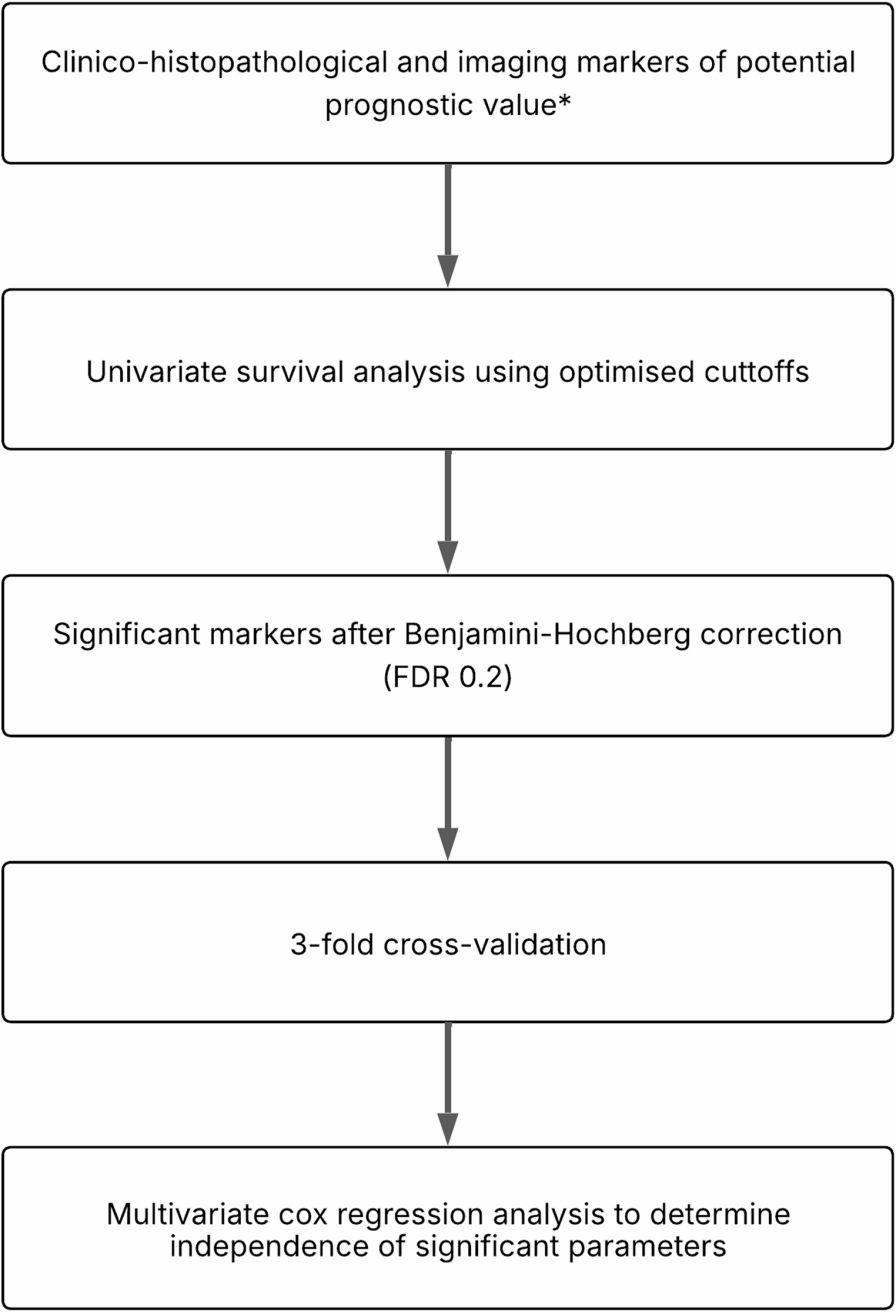
Flowchart detailing steps involved in evaluation of biomarkers (FDR = False detection rate). *Clinical markers: Patient sex, age, tumour side, location, volume, T-stage; histopathological markers: fewer than 12 lymph nodes sampled, perineural, lymphovascular or extramural invasion, tumour differentiation, tumour perforation; PET markers: SUVmax, SUVmean, TLG 40%; CT/PET texture markers: mean, standard deviation, entropy, mean of positive pixels, skewness, kurtosis

A flowchart of the statistical analysis is shown in Fig. 3. Statistical analyses were performed using SPSS version 20.0 (IBM Corp., Armonk, NY, USA), with a p-value < 0.05 considered to be significant.

## Results

In total, 421 patients were prospectively recruited between 2007 and 2019 across 9 different hospitals. From this cohort, 66 patients were identified as having stage II colorectal adenocarcinoma (41 male, 25 female, mean-age 69.1 ± 10.3yrs). 57 patients had T3N0 disease and 9 had T4N0 disease. 52 patients had colon cancer, 10 had rectal cancer, and 4 had cancer at the recto-sigmoid junction. PET and texture analysis was performed exclusively on the 66 individual primary tumours. The median tumour volume was 17.1 mL (IQR: 9.3–28.4 mL). All patients underwent primary surgical resection with curative intent. Within this stage II subpopulation, 49 patients survived to the end of the follow up period, with a mean overall survival of 88.4 months (± 42.3 months).

### Univariate Kaplan-Meier analysis

Following univariate KM analysis, multiple [^18^F]FDG PET/CT metrics were able to stratify patients into good and poor prognostic groups (Table 1). After applying Benjamini-Hochberg correction, multiple CTTA and PET parameters were found to be significant with equivalent p-values (Table 1). Pixel mean intensity at coarse texture scale (SSF = 6) was selected as the best CTTA parameter as there was a similar proportion of patients in both the good and poor prognostic groups, making it more robust and less susceptible to outlier data. Additionally, this marker was also significant at the median cutoff level (cutoff < 21.35, *p* = 0.006), making it an unbiased estimation of hazard. SUVmax was the best prognostic, significant parameter arising from the [^18^F]FDG PET/CT scan.

**Table 1:** [^18^F]FDG PET/CT imaging biomarkers associated with overall survival (FDR = False detection rate, CTTA = CT texture analysis, mpp = mean of positive pixels, pet = positron emission tomography, suv = standard uptake value, TLG 40% = Total lesion Glycolysis − 40% threshold, PTA = PET texture Analysis)

Parameter	Spatial Scale Filter (SSF)	Cutoff Value (Poor Prognosis)	Poor Prognosis Group, *n*	Good Prognosis Group, *n*	*p*-value	*p*-value post Benjamini-Hochberg Correction (20% FDR)
CTTA: mean	0	< 20.315	6	60	< 0.0001	0.0044
CTTA: kurtosis	4	>=1.405	7	59	< 0.0001	0.0089
CTTA: kurtosis	5	>=2.465	4	62	< 0.0001	0.0133
CTTA: mean	6	< 20.390	27	39	< 0.0001	0.0178
PET: SUVmax	NA	>=25.560	11	55	< 0.0001	0.0222
PET: TLG 40%	NA	>=512.915	4	62	0.0010	0.0267
PET: SUVmean	NA	>=15.090	10	56	0.0010	0.0311
CTTA: mean	5	< 18.685	30	36	0.0010	0.0356
CTTA: kurtosis	6	>=1.215	8	58	0.0010	0.0400
CTTA: mpp	0	< 28.045	4	62	0.0020	0.0444
CTTA: kurtosis	3	>=2.390	3	63	0.0020	0.0489
CTTA: mean	4	< 15.110	28	38	0.0020	0.0533
CTTA: mpp	6	< 25.655	3	63	0.0030	0.0578
CTTA: mpp	4	< 26.425	3	63	0.0040	0.0622
CTTA: mean	3	< 10.685	28	38	0.0100	0.0667
CTTA: sd	2	< 64.480	46	20	0.0140	0.0711
CTTA: skewness	3	>=0.150	12	54	0.0210	0.0756
CTTA: mpp	2	< 58.025	52	14	0.0220	0.0800
CTTA: skewness	4	<−0.780	2	62	0.0290	0.0844
CTTA: sd	0	< 27.440	49	17	0.0290	0.0889
CTTA: skewness	5	<−0.800	6	60	0.0300	0.0933
CTTA: kurtosis	0	>=0.075	34	32	0.0340	0.0978
CTTA: skewness	2	< 0.180	54	12	0.0360	0.1022
PTA: skewness	0	< 0.145	18	48	0.0400	0.1067
CTTA: mpp	3	< 28.530	4	62	0.0410	0.1111
CTTA: mean	2	< 5.165	18	48	0.0440	0.1156
CTTA: mpp	5	< 25.760	2	64	0.0490	0.1200
CTTA: skewness	6	<−1.275	2	64	0.0500	0.1244
CTTA: sd	3	< 59.925	56	10	0.0610	0.1289
CTTA: entropy	0	< 4.625	52	14	0.0660	0.1333
CTTA: sd	6	< 36.470	37	29	0.0730	0.1378
CTTA: entropy	2	< 5.210	36	30	0.0810	0.1422
CTTA: kurtosis	2	>=−0.035	40	26	0.0980	0.1467
CTTA: skewness	0	<−0.025	37	29	0.1020	0.1511
CTTA: sd	4	< 28.355	3	63	0.1080	0.1556
CTTA: entropy	5	< 4.645	14	52	0.1200	0.1600
PTA: entropy	0	>=4.370	17	49	0.1250	0.1644
PTA: sd	0	>=6725.135	46	20	0.1330	0.1689
CTTA: entropy	6	< 5.105	60	6	0.1410	0.1733
PTA: mean	0	< 4886.865	3	63	0.1630	0.1778
PTA: mpp	0	< 4886.865	3	63	0.1630	0.1822
CTTA: sd	5	< 41.505	43	23	0.1660	0.1867
CTTA: entropy	4	< 5.220	61	5	0.1860	0.1911
CTTA: entropy	3	< 5.060	40	26	0.2000	0.1956
PTA: kurtosis	0	< 0.380	62	4	0.2370	0.2000

### 3-fold cross-validation and multivariate cox regression analysis

CTTA with pixel mean intensity at coarse texture scale (SSF = 6) and SUVmax were still significant after 3-fold cross validation (*p* = 0.005 and *p* = 0.008, respectively). Multivariate analysis identified CTTA with pixel mean intensity at coarse texture scale and SUVmax as independent predictors of survival.

**Fig. 4:**
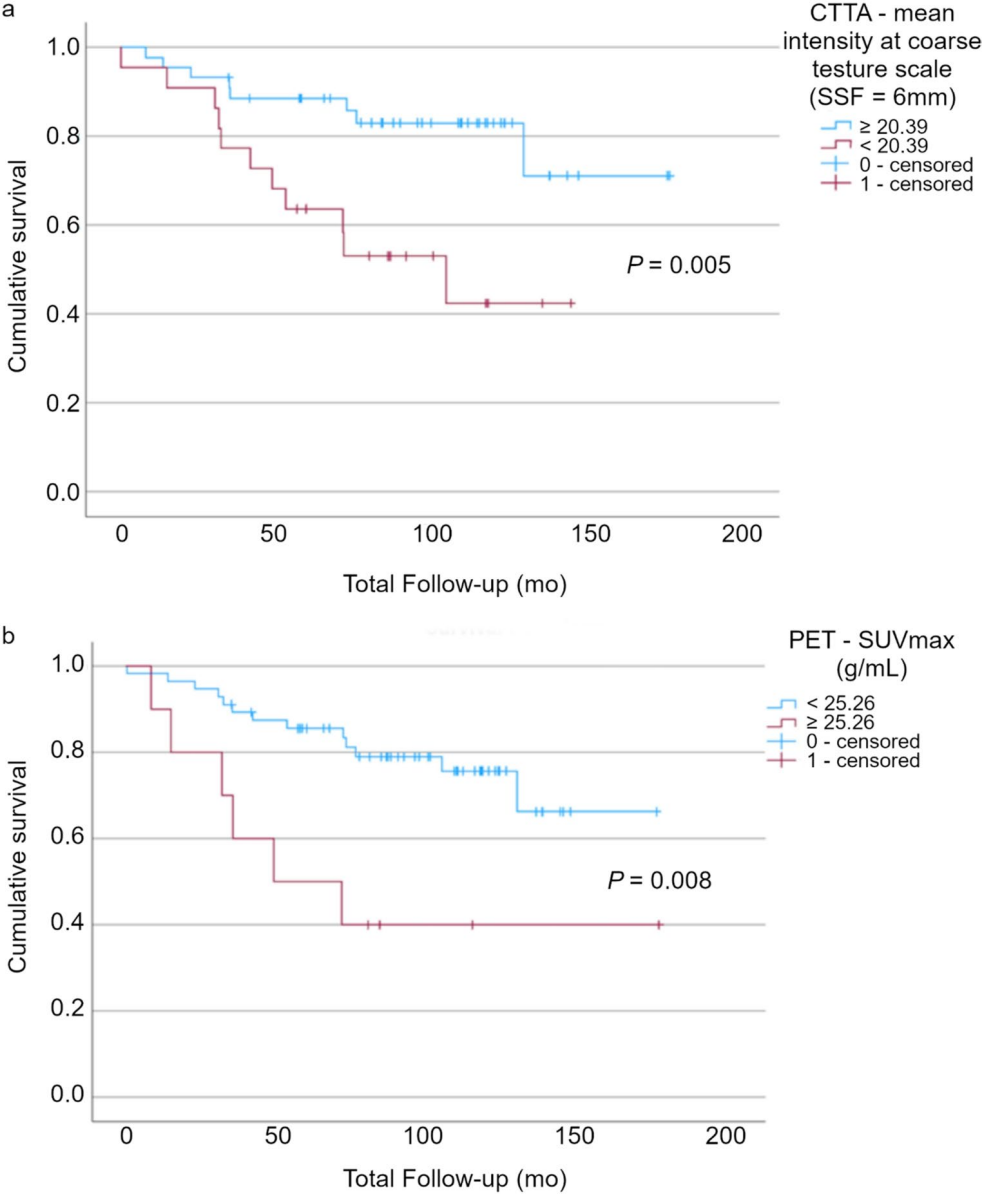
Kaplan-Meier survival analysis demonstrating (**a**) CT texture analysis with pixel mean intensity at coarse texture scale < 20.39 corresponding to higher mortality risk (*p* = 0.005); (**b**) [^18^F]FDG PET Metric SUVmax at ≥ 25.56 corresponding to higher mortality risk (*p* = 0.008)

The pixel mean intensity at coarse texture scale < 20.39 on CTTA indicates poor prognosis with a higher risk of mortality, hazard ratio: 3.40 (1.36–8.49); *p* = 0.005 (Fig. 4a). SUVmax ≥ 25.56 indicates poor prognosis with a higher risk of mortality, hazard ratio: 3.45 (1.31–9.12); *p* = 0.008 (Fig. 4b). When combined, these parameters result in a hazard ratio indicating an even higher risk of mortality in the poor prognosis group than either parameter in isolation (5 of 66 patients), hazard ratio: 5.44 (1.75–16.91); *p* = 0.001.

### Clinical and pathological variables

We performed univariate analyses on several clinical variables and found that patient sex (*p* = 0.295), patient age (*p* = 0.085), tumour laterality – left versus right side (*p* = 0.662), tumour location - rectal versus colon (*p* = 0.848) and tumour volume (*p* = 0.782), were not statistically significant predictors of overall patient survival.

We also performed univariate analyses on several high-risk pathological markers and found that the sampling of fewer than 12 lymph nodes (*n* = 3, *p* = 0.363), either perineural (*n* = 1), lymphovascular (*n* = 4) or extramural venous invasion of tumour (*n* = 12) (*p* = 0.196), poor tumour differentiation (*n* = 5, *p* = 0.372), and tumour perforation (*n* = 2, *p* = 0.475), were not statistically significant predictors of overall patient survival in our cohort.

As such, these clinical and pathological markers were excluded from the subsequent multivariate analysis.

**Fig. 5:**
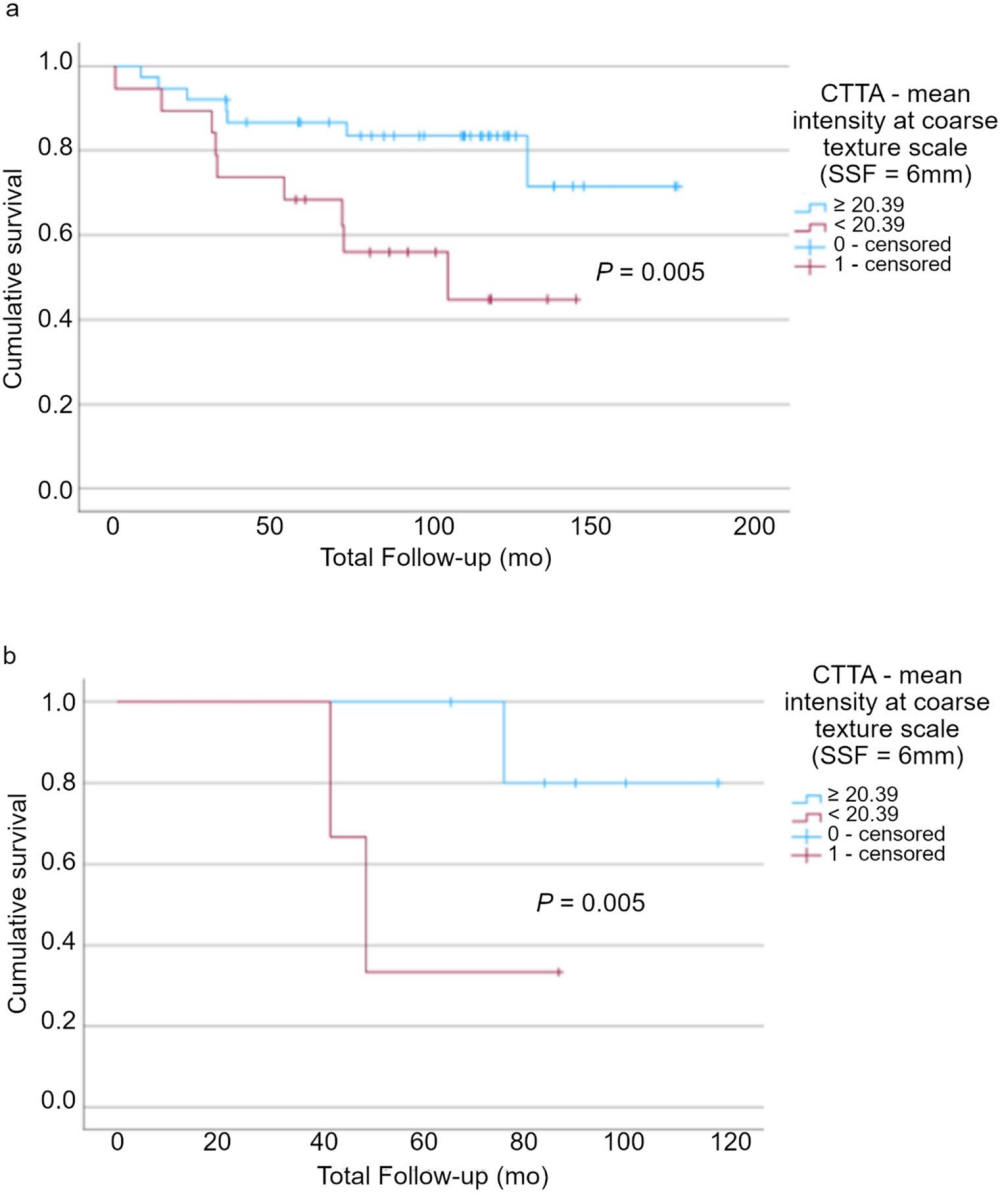
Kaplan-Meier survival analysis demonstrating CT texture analysis with pixel mean intensity at coarse texture scale < 20.39 identifies higher mortality risk in both (**a**) T3 and (**b**) T4 subgroups (*p* = 0.005)

Considering patients with T3 and T4 staging separately, the pixel mean intensity at coarse texture scale on CTTA can significantly differentiate good from poor prognosis in both groups (*p* = 0.005) (Fig. 5).

**Fig. 6:**
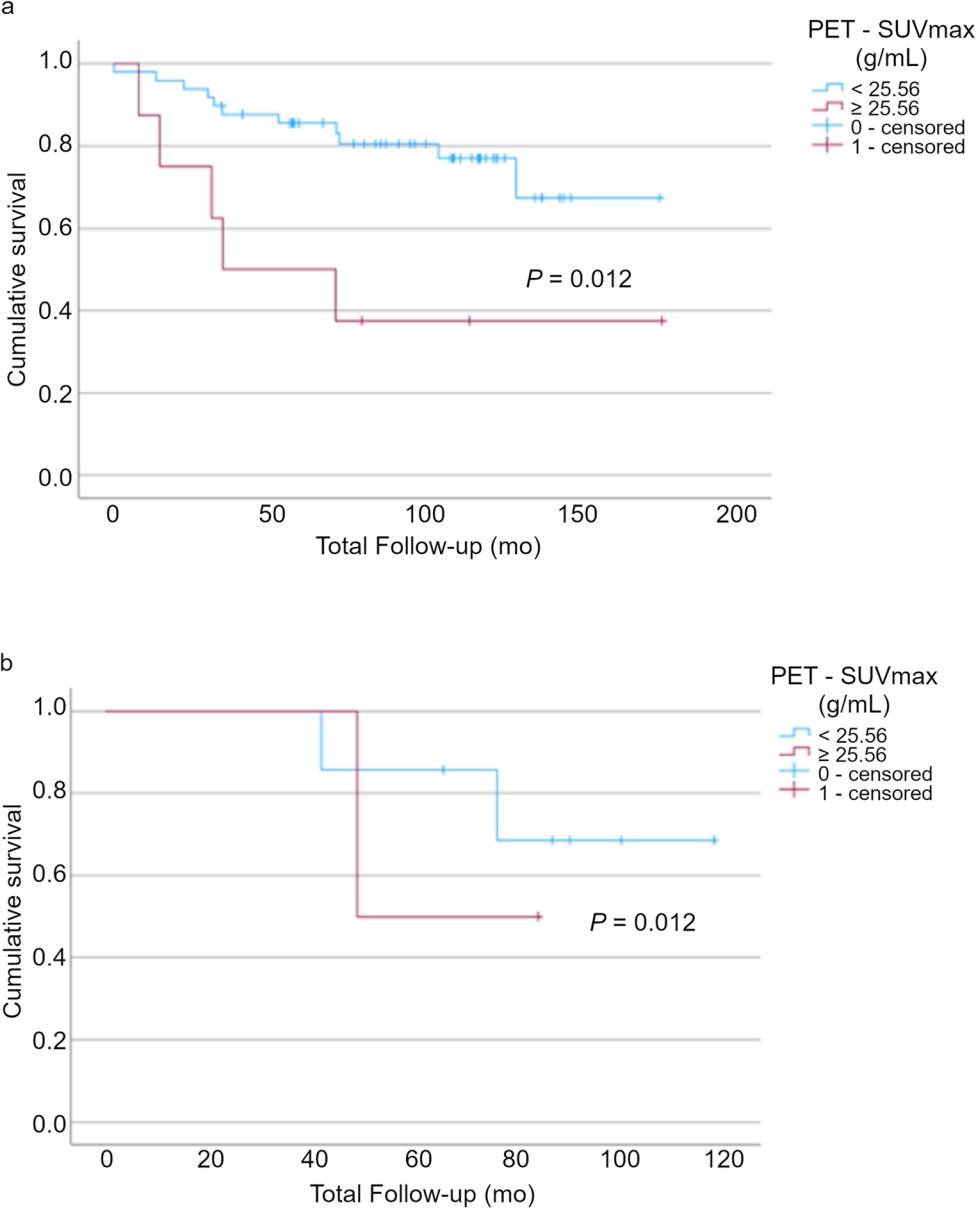
Kaplan-Meier survival analysis demonstrating SUVmax at optimised cutoff of ≥ 25.56 identifies higher mortality risk in both (**a**) T3 and (**b**) T4 subgroups (*p* = 0.012)

SUVmax can also differentiate good from poor prognosis in both groups, although differentiation is more evident at T3 stage which may be due to the smaller size of the T4 group, *n* = 9 (*p* = 0.012) (Fig. 6).

**Fig. 7:**
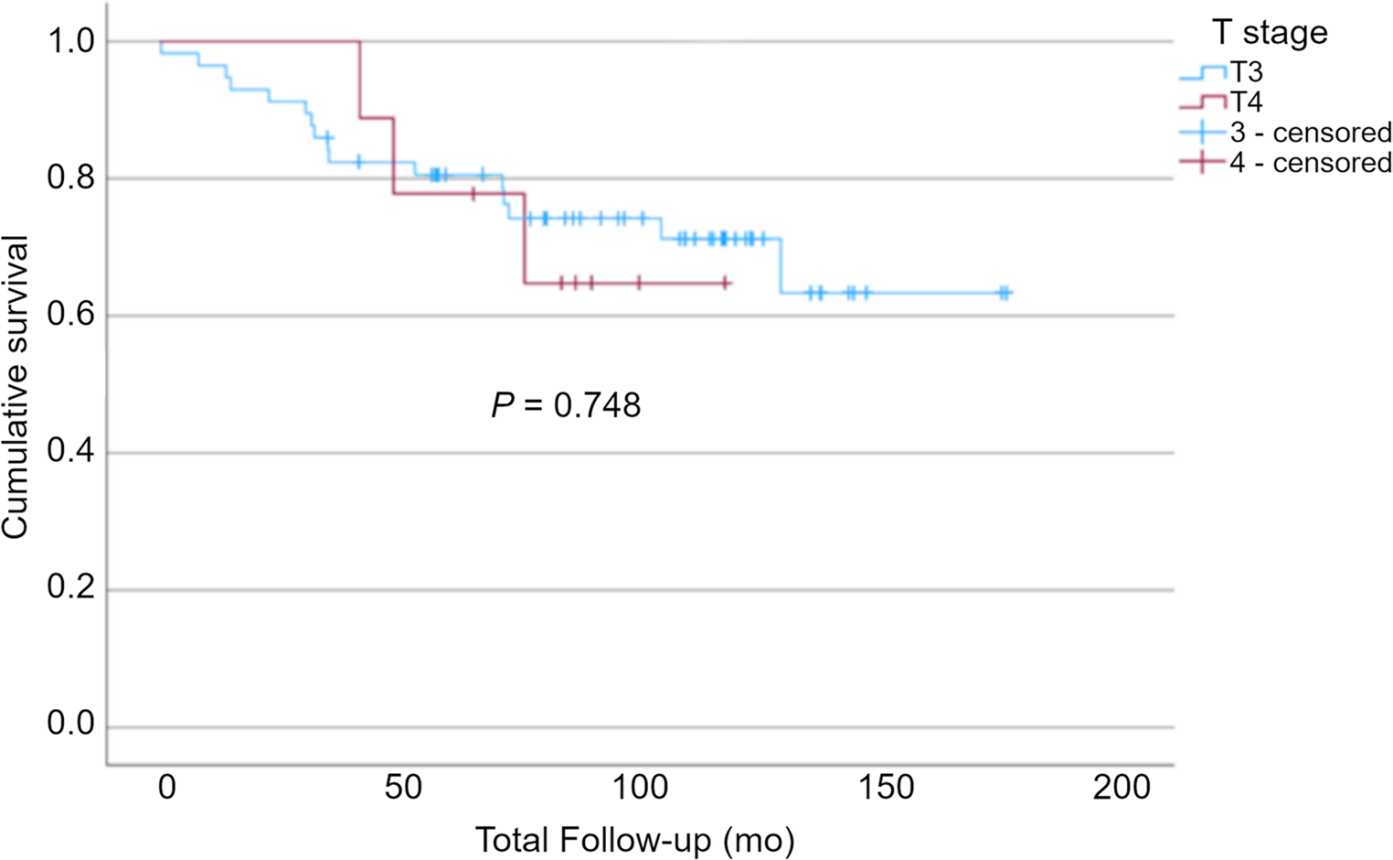
Kaplan-Meier survival analysis demonstrating no significant difference in survival between T3 and T4 disease (*p* = 0.748)

Notably, when comparing survival between the T3 and T4 groups (*p* = 0.748) (as a comparator to the imaging metrics) there is no significant difference in survival (Fig. 7).

## Discussion

Our results indicate that SUVmax is an independent prognostic marker in patients with stage II colorectal cancer. Specifically, we found that an optimal SUVmax cutoff for predicting survival was 25.56, with values above this level associated with significantly shorter survival in stage II patients. This finding is echoed in a prior study investigating survival in patients with stage I-IV colorectal cancer, which found significantly longer survival times in patients with preoperative SUVmax ≤ 11.85. The difference in cutoff value compared to our findings may be attributable to the large proportion of tumour in-situ/T1 patients included in their study (22.1%) [12]. A further study on patients with colon cancer containing a smaller proportion of stage 0 and I patients, identified an SUVmax of > 18.26 as being significantly associated with a lower 5-year disease free survival [25].

SUV on [^18^F]FDG PET/CT scans are an accepted qualitative marker of cellular metabolism, but as a quantitative measure to stratify or prognosticate disease, its utility is more questionable [26, 27]. The complex cellular and molecular mechanisms by which [^18^F]FDG accumulates in tumours are not fully understood and SUVmax values are potentially biased by physiological (blood glucose etc.), and technical factors (dose, uptake time, scan protocol etc.) including the methods around its non-standardised derivation [28]. SUVmax (potentially being based on a single pixel value) is more reproducible as a measure of ‘true’ SUV as it is less susceptible to partial volume effects than SUVmean. However, an SUVmax test-retest variability of up to 21% has been seen in a study examining the repeatability of [^18^F]FDG PET/CT scans on 62 patients with gastrointestinal malignancies [29].

Regarding technical factors, while 2D PET acquisition has inherently lower sensitivity compared with 3D mode, in the context of this study, such a disadvantage is mitigated by increased scanning time (optimised for image quality with no notable associated movement artefact), a recruitment criteria favouring larger colorectal tumours (> 2 cm), and the typically high-count nature of tumour imaging. SUV maximum and mean measurements of tumours have been shown to be consistent between 2D and 3D acquisition modes [30]. Background SUV mean measurements can be less in 3D mode due to increased noise and scatter-correction techniques, but this difference is most noticeable with shorter acquisition times (< 2 min) and would only affect tumour-to-background measurements.

In addition to SUVmax, pixel mean intensity at coarse texture scale on CTTA, was an independent prognostic marker. An optimised cutoff of 20.39 was identified, with values below this level associated with significantly shorter survival in our patient cohort. This finding is supported by previous studies including a study of patients with stage IV colorectal cancer where texture features were predictive of overall survival [17], and another study which included stage I-IV disease which found that texture features were associated with 5-year overall survival in patients with primary colorectal cancer [16]. Interestingly, when comparing survival between patients with T3N0 and T4N0 disease, we found no significant difference in overall survival. T-stage is currently suggested as one of the markers of higher-risk stage II disease and is currently used to guide treatment decisions regarding neo-adjuvant and adjuvant therapy in this group [4]. While this finding may be attributable to the mismatch in the numbers of T3/T4 patients in a relatively small study cohort, it nonetheless highlights the need for a more precise means of stratifying patients and identifying those at higher risk of recurrence. Similarly, histopathological markers that are associated with high-risk disease were not significant predictors of overall survival in this cohort.

To our knowledge this is the largest prospective study to date which aims to stratify mortality risk specifically in stage II colorectal cancer via the use of multiparametric [^18^F]FDG PET/CT. Given the projected estimate of 1.1 million deaths from colorectal cancer by 2030 [31], and the increasing incidence of the disease in younger people [32], it is imperative that new methods of risk stratification are identified to improve patient management. This objective is most pressing within the stage II group, where management is currently based largely on MDT decisions. An objective means of identifying high risk stage II patients who would benefit from neo-adjuvant or adjuvant therapies is key to improving patient outcomes. In T3N0 and T4N0 colon cancers, conflicting data is emerging regarding the use of neo-adjuvant and adjuvant chemotherapy and highlights the need for robust, non-invasive methods to risk stratify patients [33–35]. Similarly in rectal cancer, pre-operative risk stratification via quantitative imaging parameters may assist in identifying patients who are more likely to benefit from pre-operative radiotherapy and correspondingly, those in whom radiotherapy may safely be omitted [36]. The avoidance of radiotherapy and its side effects without compromising survival, has the potential to improve post treatment quality of life [6].

Conventional imaging has been shown to over stage colon cancer pre-operatively [34, 37], leading to over-treatment in patients who may in fact have pathologically lower risk colon cancer. In the FOxTROT trial, 24% of patients randomized to the control arm (up front surgery) were later found to have lower-risk stage II disease. It could be argued that participants within this group and randomised to the neoadjuvant chemotherapy arm would have received other treatment and been exposed to chemotherapy side-effects for uncertain benefit [38]. [^18^F]FDG PET/CT is currently not routinely recommended in preoperative staging of colorectal cancer [4, 6, 11], however our results highlight its potential to provide independent prognostic markers from both the [^18^F]FDG PET and CT components, thus potentially providing the MDT with additional information and a more objective means of stratifying patient risk from the outset. Additionally, improved selection of stage II patients who would (or would not) benefit from neoadjuvant and adjuvant therapies enables MDTs to make more informed, cost-effective management decisions. A Dutch, model-based evaluation has assessed cell-free circulating tumour DNA as a potential cost-effective prognostic marker in stage II colon cancer. At a current test cost of €2400, it was found that should the expense be decreased to €1500 it would offer an economical means to improve the selection of patients for adjuvant chemotherapy in stage II colon cancer [39]. The combined adverse CTTA and PET parameters in our study (5/66; HR 5.44) have a similar proportion of positive patients to the Dutch study (5/86; HR for recurrence 9.23), thus [^18^F]FDG PET/CT may prove equally cost-effective depending on the procedural cost of imaging [40].

Our study has several limitations. Despite including all eligible patients, our sample size remains relatively small. To minimize the likelihood of overfitting our data, we used the Benjamini-Hochberg procedure (controlling the false discovery rate at 0.2) to adjust p-values, and a 3-fold cross-validation of the most statistically significant texture parameters. However, larger studies will be required to validate our preliminary findings. The stage II subpopulation makes up 15.7% of our entire cohort, which is a lower proportion than reported in the literature [41]. We attribute this difference to our strict recruitment criteria requiring tumours to be larger than 2 cm, which would likely have excluded smaller stage II tumours. However, our preliminary findings have identified potential prognostic parameters and cutoff values for imaging biomarkers to justify ongoing patient recruitment and enable larger studies to take place. Further evaluation of the imaging biomarkers we have identified, in combination with additional demonstrated prognostic markers such as age and histology, could create an improved model of adequate robustness for adoption into clinical practice [42]. Our study included both rectal and colon cancer, which are known to differ in terms of pathology and prognosis [43]. We also did not adjust for the wide variety of adjuvant treatments which have the potential to significantly impact patient survival. As this was a multi-centre, observational study, patient management was determined by the local MDT in accordance with local practices and guidelines. This real-world variability in treatment implies that the prognostic markers identified from our study are robust. Future developments could include validation of a multi-variable model containing the identified prognostic imaging biomarkers in a variety of clinical settings to further refine its efficacy [42].

## Conclusion

In conclusion, within the limitation of the size of our cohort, this study demonstrates the potential for multiparametric [^18^F]FDG PET/CT to provide prognostic imaging markers in patients with stage II colorectal cancer over and above traditional means of risk stratifying such as T-stage. The ability to risk-stratify patients from the outset allows for the selection of the most appropriate therapeutic strategies and enables a personalised, cost-effective approach.

## Data Availability

The datasets generated and analysed during the current study are not publicly available in order to protect participant privacy. However, anonymised data may be made available from the corresponding author upon reasonable request.

## References

[1] Arnold M, Sierra MS, Laversanne M, Soerjomataram I, Jemal A, Bray F. Global patterns and trends in colorectal cancer incidence and mortality. Gut. 2017;66(4):683–91. 10.1136/gutjnl-2015-310912. 26818619 10.1136/gutjnl-2015-310912

[2] Bray F, Ferlay J, Soerjomataram I, Siegel RL, Torre LA, Jemal A. Global cancer statistics 2018: GLOBOCAN estimates of incidence and mortality worldwide for 36 cancers in 185 countries. CA Cancer J Clin. 2018;68(6):394–424. 10.3322/caac.21492. 30207593 10.3322/caac.21492

[3] Giannakis M, Ng K. To treat or not to treat: adjuvant therapy for stage II colon cancer in the era of precision oncology. J Oncol Pract. 2017;13(4):242–4. 10.1200/JOP.2017.022103. 28399380 10.1200/JOP.2017.022103

[4] Argilés G, Tabernero J, Labianca R, et al. Localised colon cancer: ESMO clinical practice guidelines for diagnosis, treatment and follow-up†. Ann Oncol. 2020;31(10):1291–305. 10.1016/j.annonc.2020.06.022. 32702383 10.1016/j.annonc.2020.06.022

[5] Gollins S, Moran B, Adams R, Association of coloproctology of great Britain & Ireland (ACPGBI), et al. Association of Coloproctology of Great Britain & Ireland (<scp>ACPGBI</scp>): Guidelines for the Management of Cancer of the Colon, Rectum and Anus (2017) – Multidisciplinary Management. Colorectal Dis. 2017;19(S1):37–66. 10.1111/codi.13705. 10.1111/codi.13705 28632307

[6] Glynne-Jones R, Wyrwicz L, Tiret E, et al. Rectal cancer: ESMO clinical practice guidelines for diagnosis, treatment and follow-up. Ann Oncol. 2017;28:iv22–40. 10.1093/annonc/mdx224. 28881920 10.1093/annonc/mdx224

[7] Böckelman C, Engelmann BE, Kaprio T, Hansen TF, Glimelius B. Risk of recurrence in patients with colon cancer stage II and III: a systematic review and meta-analysis of recent literature. Acta Oncol. 2015;54(1):5–16. 10.3109/0284186X.2014.975839. 25430983 10.3109/0284186X.2014.975839

[8] André T, de Gramont A, Vernerey D, et al. Adjuvant fluorouracil, leucovorin, and oxaliplatin in stage II to III colon cancer: updated 10-year survival and outcomes according to BRAF mutation and mismatch repair status of the MOSAIC study. J Clin Oncol. 2015;33(35):4176–87. 10.1200/JCO.2015.63.4238. 26527776 10.1200/JCO.2015.63.4238

[9] O’Connor ES, Greenblatt DY, LoConte NK, et al. Adjuvant chemotherapy for stage II colon cancer with poor prognostic features. J Clin Oncol. 2011;29(25):3381–8. 10.1200/JCO.2010.34.3426. 21788561 10.1200/JCO.2010.34.3426 PMC3164243

[10] Costas-Chavarri A, Nandakumar G, Temin S, et al. Treatment of patients with Early-Stage colorectal cancer: ASCO Resource-Stratified guideline. J Glob Oncol. 2019;5:1–19. 10.1200/JGO.18.00214. 30802158 10.1200/JGO.18.00214 PMC6426503

[11] Maffione AM, Rubello D, Caroli P, Colletti PM, Matteucci F. Is it time to introduce PET/CT in colon cancer guidelines?? Clin Nucl Med. 2020;45(7):525–30. 10.1097/RLU.0000000000003076. 32433179 10.1097/RLU.0000000000003076

[12] Shi D, Cai G, Peng J, et al. The preoperative SUVmax for (18)F-FDG uptake predicts survival in patients with colorectal cancer. BMC Cancer. 2015;15:991. 10.1186/s12885-015-1991-5. 26689966 10.1186/s12885-015-1991-5 PMC4687154

[13] Xu J, Li Y, Hu S, Lu L, Gao Z, Yuan H. The significant value of predicting prognosis in patients with colorectal cancer using 18F-FDG PET metabolic parameters of primary tumors and hematological parameters. Ann Nucl Med. 2019;33(1):32–8. 10.1007/s12149-018-1299-z. 30218280 10.1007/s12149-018-1299-z

[14] Alrahawy M, Aker M, Issa M, et al. Textural analysis as a predictive biomarker in rectal cancer. Cureus. 2022;14(12):e32241. 10.7759/cureus.32241. 36620843 10.7759/cureus.32241 PMC9813797

[15] Ganeshan B, Goh V, Mandeville HC, Ng QS, Hoskin PJ, Miles KA. Non-small cell lung cancer: histopathologic correlates for texture parameters at CT. Radiology. 2013;266(1):326–36. 10.1148/radiol.12112428. 23169792 10.1148/radiol.12112428

[16] Ng F, Ganeshan B, Kozarski R, Miles KA, Goh V. Assessment of primary colorectal cancer heterogeneity by using whole-tumor texture analysis: contrast-enhanced CT texture as a biomarker of 5-year survival. Radiology. 2013;266(1):177–84. 10.1148/radiol.12120254. 23151829 10.1148/radiol.12120254

[17] Negreros-Osuna AA, Parakh A, Corcoran RB, et al. Radiomics texture features in advanced colorectal cancer: correlation with BRAF mutation and 5-year overall survival. Radiology: Imaging Cancer. 2020;2(5):e190084. 10.1148/rycan.2020190084. 33778733 10.1148/rycan.2020190084 PMC7983710

[18] Chen C, Ou X, Li H, et al. Contrast-enhanced CT texture analysis: a new set of predictive factors for small cell lung cancer. Mol Imaging Biol. 2020;22(3):745–51. 10.1007/s11307-019-01419-1. 31429049 10.1007/s11307-019-01419-1

[19] Brenet Defour L, Mulé S, Tenenhaus A, et al. Hepatocellular carcinoma: CT texture analysis as a predictor of survival after surgical resection. Eur Radiol. 2019;29(3):1231–9. 10.1007/s00330-018-5679-5. 30159621 10.1007/s00330-018-5679-5

[20] Zhang Y, Li X, Lv Y, Gu X. Review of value of CT texture analysis and machine learning in differentiating Fat-Poor renal angiomyolipoma from renal cell carcinoma. Tomography. 2020;6(4):325–32. 10.18383/j.tom.2020.00039. 33364422 10.18383/j.tom.2020.00039 PMC7744193

[21] Atkinson C, Ganeshan B, Endozo R, et al. Radiomics-based texture analysis of ^68^Ga-DOTATATE positron emission tomography and computed tomography images as a prognostic biomarker in adults with neuroendocrine cancers treated with ^177^Lu-DOTATATE. Front Oncol. 2021;11:686235. 10.3389/fonc.2021.686235. 34408979 10.3389/fonc.2021.686235 PMC8366561

[22] Song SE, Seo BK, Cho KR, et al. Prediction of inflammatory breast cancer survival outcomes using computed tomography-based texture analysis. Front Bioeng Biotechnol. 2021;9:695305. 10.3389/fbioe.2021.695305. 34354986 10.3389/fbioe.2021.695305 PMC8329959

[23] Miles KA, Ganeshan B, Hayball MP. CT texture analysis using the filtration-histogram method: what do the measurements mean? Cancer Imaging. 2013;13(3):400–6. 10.1102/1470-7330.2013.9045. 24061266 10.1102/1470-7330.2013.9045 PMC3781643

[24] Chen K, Collins G, Wang H, Toh JWT. Pathological features and prognostication in colorectal cancer. Curr Oncol. 2021;28(6):5356–83. 10.3390/curroncol28060447. 34940086 10.3390/curroncol28060447 PMC8700531

[25] Li D, Wang Y, Liu W, et al. The correlation between ^18^F-FDG PET/CT imaging SUVmax of preoperative colon cancer primary lesions and clinicopathological factors. J Oncol. 2021;2021:4312296. 10.1155/2021/4312296. 34567115 10.1155/2021/4312296 PMC8463203

[26] Ahn H, Won Lee J, Jang SH, et al. Prognostic significance of imaging features of peritumoral adipose tissue in FDG PET/CT of patients with colorectal cancer. Eur J Radiol. 2021;145:110047. 10.1016/j.ejrad.2021.110047. 34801879 10.1016/j.ejrad.2021.110047

[27] Yin YX, Xie MZ, Liang XQ, Ye ML, Li JL, Hu BL. Clinical significance and prognostic value of the maximum standardized uptake value of ^18^F-flurodeoxyglucose positron emission tomography-computed tomography in colorectal cancer. Front Oncol. 2021;11:741612. 10.3389/fonc.2021.741612. 34956868 10.3389/fonc.2021.741612 PMC8695495

[28] Huang J, Huang L, Zhou J, et al. Elevated tumor-to-liver uptake ratio (TLR) from ^18^F-FDG-PET/CT predicts poor prognosis in stage IIA colorectal cancer following curative resection. Eur J Nucl Med Mol Imaging. 2017;44(12):1958–68. 10.1007/s00259-017-3779-0. 28812134 10.1007/s00259-017-3779-0 PMC5656694

[29] Velasquez LM, Boellaard R, Kollia G, et al. Repeatability of 18F-FDG PET in a multicenter phase I study of patients with advanced gastrointestinal malignancies. J Nucl Med. 2009;50(10):1646–54. 10.2967/jnumed.109.063347. 19759105 10.2967/jnumed.109.063347

[30] Komar G, Teräs M, Seppänen M, et al. Comparison of 2D and 3D performance for FDG PET with different acquisition times in oncological patients. Nucl Med Commun. 2009;30(1):16–24. 10.1097/mnm.0b013e328315a22a. 19306510 10.1097/mnm.0b013e328315a22a

[31] Sawicki T, Ruszkowska M, Danielewicz A, Niedźwiedzka E, Arłukowicz T, Przybyłowicz KE. A review of colorectal cancer in terms of epidemiology, risk factors, development, symptoms and diagnosis. Cancers (Basel). 2021. 10.3390/cancers13092025. 33922197 10.3390/cancers13092025 PMC8122718

[32] Wong MCS, Huang J, Lok V, et al. Differences in incidence and mortality trends of colorectal cancer worldwide based on sex, age, and anatomic location. Clin Gastroenterol Hepatol. 2021;19(5):955–e96661. 10.1016/j.cgh.2020.02.026. 32088300 10.1016/j.cgh.2020.02.026

[33] Chalabi M, Fanchi LF, Dijkstra KK, et al. Neoadjuvant immunotherapy leads to pathological responses in MMR-proficient and MMR-deficient early-stage colon cancers. Nat Med. 2020;26(4):566–76. 10.1038/s41591-020-0805-8. 32251400 10.1038/s41591-020-0805-8

[34] Karoui M, Rullier A, Piessen G, et al. Perioperative FOLFOX 4 versus FOLFOX 4 plus cetuximab versus immediate surgery for High-Risk stage II and III colon cancers: a phase II multicenter randomized controlled trial (PRODIGE 22). Ann Surg. 2020;271(4):637–45. 10.1097/SLA.0000000000003454. 31356278 10.1097/SLA.0000000000003454

[35] Weinberg BA, Sackstein PE, Yu J, et al. Evolving standards of care in the management of localized colorectal cancer. Am Soc Clin Oncol Educ Book. 2024;44(3):e432034. 10.1200/EDBK_432034. 38768426 10.1200/EDBK_432034

[36] Sauer R, Liersch T, Merkel S, et al. Preoperative versus postoperative chemoradiotherapy for locally advanced rectal cancer: results of the German CAO/ARO/AIO-94 randomized phase III trial after a median follow-up of 11 years. J Clin Oncol. 2012;30(16):1926–33. 10.1200/JCO.2011.40.1836. 22529255 10.1200/JCO.2011.40.1836

[37] Platt JR, Ansett J, Seligmann JF, West NP, Tolan DJM. The impact of mismatch repair status and systemic inflammatory markers on radiological staging in colon cancer. Br J Radiol. 2023;96(1150):20230098. 10.1259/bjr.20230098. 37493144 10.1259/bjr.20230098 PMC10546445

[38] Foxtrot Collaborative Group. Feasibility of preoperative chemotherapy for locally advanced, operable colon cancer: the pilot phase of a randomised controlled trial. Lancet Oncol. 2012;13(11):1152–60. 10.1016/S1470-2045(12)70348-0. 23017669 10.1016/S1470-2045(12)70348-0 PMC3488188

[39] Kramer A, Greuter MJE, Schraa SJ, et al. Early evaluation of the effectiveness and cost-effectiveness of ctDNA-guided selection for adjuvant chemotherapy in stage II colon cancer. Ther Adv Med Oncol. 2024;16:17588359241266164. 10.1177/17588359241266164. 39175989 10.1177/17588359241266164 PMC11339739

[40] Smith AF, Hall PS, Hulme CT, et al. Cost-effectiveness analysis of PET-CT-guided management for locally advanced head and neck cancer. Eur J Cancer. 2017;85:6–14. 10.1016/j.ejca.2017.07.054. 28881249 10.1016/j.ejca.2017.07.054

[41] Carsin AE, Sharp L, Cronin-Fenton DP, Céilleachair AO, Comber H. Inequity in colorectal cancer treatment and outcomes: a population-based study. Br J Cancer. 2008;99(2):266–74. 10.1038/sj.bjc.6604467. 18594530 10.1038/sj.bjc.6604467 PMC2480963

[42] Halligan S, Menu Y, Mallett S. Why did European radiology reject my radiomic biomarker paper? How to correctly evaluate imaging biomarkers in a clinical setting. Eur Radiol. 2021;31(12):9361–8. 10.1007/s00330-021-07971-1. 34003349 10.1007/s00330-021-07971-1 PMC8589811

[43] Li JN, Zhao L, Wu J, et al. Differences in gene expression profiles and carcinogenesis pathways between colon and rectal cancer. J Dig Dis. 2012;13(1):24–32. 10.1111/j.1751-2980.2011.00551.x. 22188913 10.1111/j.1751-2980.2011.00551.x

